# Variable RBE in proton therapy: comparison of different model predictions and their influence on clinical-like scenarios

**DOI:** 10.1186/s13014-016-0642-6

**Published:** 2016-05-17

**Authors:** Giulia Giovannini, Till Böhlen, Gonzalo Cabal, Julia Bauer, Thomas Tessonnier, Kathrin Frey, Jürgen Debus, Andrea Mairani, Katia Parodi

**Affiliations:** Ludwig-Maximilians-Universität München, Am Coulombwall 1, D-85748 Garching b. München, Germany; University of Pavia, Department of Physics, Via Bassi 6, I-27100 Pavia, Italy; Department of Radiation Oncology, Heidelberg University Clinic, Im Neuenheimer, Feld 400, D-69120 Heidelberg, Germany; European Organization for Nuclear Research CERN, CH-1211, Geneva, 23 Switzerland; Heidelberg Ion Beam Therapy Center, Im Neuenheimer Feld 450, D-69120 Heidelberg, Germany; Medical Physics Unit, CNAO Foundation, Via Strada Campeggi 53, I-27100 Pavia, Italy; Now with Medical Physics Division, EBG MedAustron GmbH, Marie Curie-Straβe 5, Wiener Neustadt, A-2700 Austria

**Keywords:** Proton therapy, Relative biological effectiveness, Monte Carlo, FLUKA

## Abstract

**Background:**

In proton radiation therapy a constant relative biological effectiveness (RBE) of 1.1 is usually assumed. However, biological experiments have evidenced RBE dependencies on dose level, proton linear energy transfer (LET) and tissue type. This work compares the predictions of three of the main radio-biological models proposed in the literature by Carabe-Fernandez, Wedenberg, Scholz and coworkers.

**Methods:**

Using the chosen models, a spread-out Bragg peak (SOBP) as well as two exemplary clinical cases (single field and two fields) for cranial proton irradiation, all delivered with state-of-the-art pencil-beam scanning, have been analyzed in terms of absorbed dose, dose-averaged LET (LET_*D*_), RBE-weighted dose (*D*_RBE_) and biological range shift distributions.

**Results:**

In the systematic comparison of RBE predictions by the three models we could show different levels of agreement depending on (*α*/*β*)_*x*_ and LET values. The SOBP study emphasizes the variation of LET_*D*_ and RBE not only as a function of depth but also of lateral distance from the central beam axis. Application to clinical-like scenario shows consistent discrepancies from the values obtained for a constant RBE of 1.1, when using a variable RBE scheme for proton irradiation in tissues with low (*α*/*β*)_*x*_, regardless of the model. Biological range shifts of 0.6– 2.4 mm (for high (*α*/*β*)_*x*_) and 3.0 – 5.4 mm (for low (*α*/*β*)_*x*_) were found from the fall-off analysis of individual profiles of RBE-weighted fraction dose along the beam penetration depth.

**Conclusions:**

Although more experimental evidence is needed to validate the accuracy of the investigated models and their input parameters, their consistent trend suggests that their main RBE dependencies (dose, LET and (*α*/*β*)_*x*_) should be included in treatment planning systems. In particular, our results suggest that simpler models based on the linear-quadratic formalism and LET_D_ might already be sufficient to reproduce important RBE dependencies for re-evaluation of plans optimized with the current RBE = 1.1 approximation. This approach would be a first step forward to consider RBE variations in proton therapy, thus enabling a more robust choice of biological dose delivery. The latter could in turn impact clinical outcome, especially in terms of reduced toxicities for tumors adjacent to organs at risk.

## Background

Light ion beams exhibit favorable physical characteristics, which allow for a highly conformal and biologically effective dose delivery to the tumor, while optimally sparing adjacent normal tissue. This rationale has boosted their application in radiation therapy. For clinical patient treatment, a constant relative biological effectiveness (RBE) of 1.1 is currently recommended and applied for proton beams [1], despite the fact that the RBE of protons depends on many factors such as dose level, linear-energy transfer (LET), tissue radio-sensitivity, oxygen concentration and biological end-point [2, 3]. Using a constant RBE value at each position of a spread-out Bragg peak (SOBP) of a proton beam and within every tissue is an approximation, generally supported by the fact that the available biological data are insufficient to justify clinical usage of other proposed approaches [4]. In a recent review [3], Paganetti analysed a large amount of experimental data available from published literature. His work highlighted that there is a trend of an increase in RBE as (α/β)_x_ (i.e., the ratio between the linear and the quadratic term of the linear quadratic (LQ) model [5] for the reference photon radiation) decreases and as the dose decreases. This dose-effect has been seen especially for systems with low (α/β)_x_.

Several phenomenological models for RBE predictions have been proposed, for instance by Wilkens and Oelfke [6], Tilly et al. [7], Carabe-Fernandez et al. [8], and Wedenberg et al. [9]. Biophysical models are available as well, such as the microdosimetric-kinetic-model (MKM, [10]), the local effect model (LEM, [11, 12]), and the repair-misrepair-fixation (RMF) model [13]. In this work, the models by Carabe-Fernandez et al., Wedenberg et al. and a re-implementation of the LEM were chosen for three main reasons:

- Both biophysical (LEM) and phenomenological (Carabe-Fernandez et al. and Wedenberg et al.) models have been taken into account. In terms of biophysical models, LEM is the model already used by treatment planning systems of European dual ion (proton and carbon ion) therapy facilities for biological optimization in carbon ion therapy.
- The three selected models assume or predict different trends of the LET dependence for the β parameter of the LQ model. For an increasing LET, β is decreasing according to the implemented version of the LEM, increasing in Carabe et al., and constant in Wedenberg et al.
- Phenomenological models as simple as possible with no plan-dependent parameters were chosen to make the comparison more straightforward. For this reason, the Wilkens and Oelfke model [6] has been excluded from the analysis.

The increase of RBE with depth of a proton beam causes a change of the biological beam range. Carabe and collaborators [4] have quantified the range shift for a SOBP in water and for a clinical case applying the Carabe-Fernandez et al. model. They have found that the shift increases with the physical range but decreases with increasing dose or (α/β)_x_.

More recently, Grün and collaborators [14] have studied the impact of the beam energy, tissue type and dose level on the biological range of the proton beams by applying the LEM and performing calculations for SOBPs in water. They have found that the biological range of proton beams is strongly dependent on the physical properties of the beam as well as on absorbed dose and the biological properties of the irradiated tissue. Extensions in depth of the biologically effective SOBP up to 4 mm (with respect to the value obtained using a constant RBE of 1.1) have been found.

Treatment planning studies using a variable RBE scheme have been performed in the past. Tilly and collaborators have studied the influence of RBE variations in a clinical proton treatment for hypopharinx cancer [7] applying their biological model. They have shown the importance of considering RBE corrections especially when organs at risk (OARs) are located immediately behind the target volume. Carabe and collaborators [15] have applied their model to study the clinical consequences of RBE variations in proton therapy of the prostate, brain and liver. They have found that, for standard fractionated regimens, RBE values larger than 1.1 were encountered in prostate and liver tumors as well as the OARs in the brain. Conversely, RBE values lower than 1.1 have typically been observed in hypofractionated regimes. Wedenberg and Toma-Dasu [16] have applied the Wedenberg et al. model for three brain cases irradiated with proton beams. They have found that disregarding RBE variations might lead to suboptimal proton plans (lower biological dose in the target and higher biological dose in the normal tissues).

In this work, we compare the predictions of three selected radio-biological models for two tissue specific parameters characterized by a ratio (α/β)_x_ = 2 Gy and 10 Gy for the photon reference radiation. The (α/β)_x_ values have been chosen to represent late-responding tissues (low (α/β)_x_ around 2–3 Gy) and early-responding normal tissue and most common tumors (high (α/β)_x_ around 10 Gy). We study in depth the impact of the different model predictions on RBE, RBE-weighted dose and biological range for two exemplary clinical cranial irradiations (single field and two fields configuration) with state-of-the-art pencil-beam scanning. Moreover, a SOBP has been simulated in water to study depth- and lateral- dependent biological quantities for a tissue with (α/β)_x_ = 2 Gy in a well-controlled scenario without tissue heterogeneities. The head-to-head comparisons of the different RBE models for the same clinical cases allow assessment of RBE variations and the impact of the choice of the RBE model, revealing also some common trends in the predictions of variable RBE schemes in comparison to the current approximation of a constant (equal 1.1) RBE. The latter is important to evaluate the performance of simpler phenomenological models for a straightforward implementation in treatment planning systems (TPS), compared to more complex biophysical models. Moreover, our work highlights the relevance of LET variations not only along the beam penetration depth, but also for transversal profiles. Overall, our findings can be used to support decision-making processes in the clinical practice keeping in mind the uncertainties of the biological data and the observed spread of model predictions. In particular, we strongly encourage the next generation of TPSs to include RBE-based calculations for proton radiation therapy, even only with the simpler phenomenological models, to enable comparisons between the standard RBE 1.1 approach and a variable RBE scheme for improved robustness of the final treatment plan.

## Methods

### Modeling the biological effectiveness of protons

#### The Carabe-Fernandez et al. model

The first of the two phenomenological models chosen for the comparison is the extension by Carabe-Fernandez et al. [8] of the approach proposed by Dale and Jones [17]. The aim of the approach is to determine, within the LQ model, relationships between the parameters α and β for proton radiation and the *α*_*x*_ and *β*_*x*_ for photon radiation. Within the LQ framework, considering that a proton absorbed dose *D* and a photon dose *D*_*x*_ are isoeffective if:

$$ \alpha \kern0.1em D+\beta \kern0.1em {D}^2={\alpha}_x\kern0.1em {D}_x+{\beta}_x\kern0.1em {D}_x^2, $$

Dividing by *D*_*x*_ and considering that RBE = *D*_*x*_*/D* by definition, thus expressing *D* as *D*_*x*_*/RBE,* we arrive at:

$$ \left({\alpha}_x+{\beta}_x\kern0.1em {D}_x\right)\kern0.1em {\mathrm{RBE}}^2-\alpha \kern0.1em \mathrm{R}\mathrm{B}\mathrm{E}-\beta\ {D}_x=0. $$

Solving for the positive value of RBE:

1 $$ \mathrm{R}\mathrm{B}\mathrm{E}\left(\alpha, \beta, {\alpha}_x,{\beta}_x,{D}_x\right)=\frac{\alpha +\sqrt{\alpha^2+4\beta \kern0.1em {D}_x\left({\alpha}_x+{\beta}_x\kern0.1em {D}_x\right)}}{2\kern0.1em \left({\alpha}_x+{\beta}_x\kern0.1em {D}_x\right)}. $$

Then, two quantities are defined:

2 $$ {\mathrm{RBE}}_{\max}\equiv \frac{\alpha }{\alpha_x}, $$

3 $$ {\mathrm{RBE}}_{\min}\equiv \sqrt{\frac{\beta }{\beta_x}}. $$

RBE_max_ and RBE_min_ correspond to the asymptotic values of the RBE at *D =* 0 and *D =* ∞, respectively. They are assumed to contain the dependence of the RBE on LET. Using Eqs. (1), (2) and (3), an expression for the RBE that only depends on photon LQ parameters and the photon dose is obtained:

4 $$ \mathrm{R}\mathrm{B}\mathrm{E}\left({\alpha}_x,{\beta}_x,{D}_x\right)=\frac{{\left(\frac{\alpha }{\beta}\right)}_x\kern0.1em {\mathrm{RBE}}_{\max }+\sqrt{{\left(\frac{\alpha }{\beta}\right)}_x^2\kern0.1em {\mathrm{RBE}}_{\max}^2+4{D}_x\left[{\left(\frac{\alpha }{\beta}\right)}_x+{D}_x\right]\kern0.1em {\mathrm{RBE}}_{\min}^2}}{2\kern0.1em \left[{\left(\frac{\alpha }{\beta}\right)}_x+{D}_x\right]}. $$

Four sets of experimental data for V79 cells were used by the authors to assess the dependence of RBE_max_ and RBE_min_ on the dose-averaged LET (LET_*D*_). From the linear regression analysis, the authors calculated intersection points and slopes of the linear fit. Then, for V79 cells a (*α*/*β*)_*x*_ value of 2.686 Gy is obtained by averaging over all the reported experimental values in each data set. A reciprocal dependence of RBE_max_ and RBE_min_on (*α*/*β*)_*x*_ is assumed. This means that for those tissues with (*α*/*β*)_*x*_ = 2.686 Gy the slope must be exactly that same one found fitting V79 cells data, whereas for other tissues the slope must increase for decreasing (*α*/*β*)_*x*_ and vice versa:

5 $$ {\mathrm{RBE}}_{\max}\left[{\mathrm{LET}}_D,{\left(\alpha /\beta \right)}_x\right]=0.834+0.154\cdot \frac{2.686}{{\left(\alpha /\beta \right)}_x}\;{\mathrm{LET}}_D, $$

6 $$ {\mathrm{RBE}}_{\min}\left[{\mathrm{LET}}_D,{\left(\alpha /\beta \right)}_x\right]=1.09+0.006\cdot \frac{2.686}{{\left(\alpha /\beta \right)}_x}\;{\mathrm{LET}}_D. $$

For the comparison of Carabe-Fernandez model predictions against the other models we have used the unrestricted LET in water instead of LET_*D*_ used in the original model which means that cells are represented by water and each particle has the same LET (corresponding to in vitro irradiation with mono-energetic protons). Equation (4) can be employed in combination with Eqs. (5) and (6) for studying the RBE of protons in human tissues, noting that the model has been derived using V79 cell data. We will refer in the next sections to the Carabe-Fernandez et al. model as CAR model.

#### The Wedenberg et al. model

The second considered model has been developed by Wedenberg et al. [9]. Despite its simple formalism, this model succeeds in capturing the basic features of RBE for protons with minimum well validated assumptions. First, *α* is assumed to vary linearly with the LET and to approach *α*_*x*_ when the LET decreases in the LET range of clinical interest in proton therapy:

7 $$ \frac{\alpha }{\alpha_x}=1+k\cdot \kern0.62em \mathrm{LET}. $$

Since the ratio *α/α*_*x*_ is known to decrease with increasing LET after 30 keV/μm, Eq. (7) is supposed to be valid for LET lower than this value [9]. Second, since different values of the *α/α*_*x*_ ratio for similar LET values have been reported in several studies and this is supposed to be due to the differences between cell lines, an inverse relationship between the slope *k* and the tissue response ratio (*α*/*β*)_*x*_ is assumed:

8 $$ \frac{\alpha }{\alpha_x}=1+\frac{q}{{\left(\alpha /\beta \right)}_x}\cdot \kern0.62em \mathrm{LET}. $$

*q* is a free parameter which does not depend on the physical characteristics of the proton beam and on the biological system. Its value has been determined as 0.434 (Gy μm)/keV fitting the experimental data reported in [9]. In other words, LET variations affect the survival of low (*α*/*β*)_*x*_ ratio tissues more than high ratio ones. In the Wedenberg et al. approach, *β* is assumed to be LET independent and is merely assumed to be:

9 $$ \beta ={\beta}_x. $$

If we use the same formalism as introduced for the Carabe-Fernandez et al. model we obtain for the Wedenberg et al. model:

10 $$ {\mathrm{RBE}}_{\max}\left[\mathrm{LET},{\left(\alpha /\beta \right)}_x\right]=1.00+\frac{0.434}{{\left(\alpha /\beta \right)}_x}\;\mathrm{LET}, $$

11 $$ {\mathrm{RBE}}_{\min }=1.0. $$

Each hypothesis of the model has been statistically tested on the basis of an experimental data set, including 10 different cell lines with (*α*/*β*)_*x*_ values ranging from 2.7 Gy up to more than 70 Gy, irradiated with proton beams with LET values ranging from 6 keV/μm up to 30 keV/μm. According to the authors, the model is able to predict the RBE based on the delivered dose, the LET, and the tissue specific parameter (*α*/*β*)_*x*_ of photons. For MC-based patient calculations we have applied the Wedenberg et al. formalism using the LET_*D*_ instead of the LET, i. e. taking into account the produced mixed radiation field produced. We will refer in the next sections to the Wedenberg et al. model as WED model.

#### The local effect model-version IV

The LEM-version IV developed by GSI Helmholtzzentrum für Schwerionenforschung [11, 12] relates the biological response directly to the double-strand breaks (DSB) pattern. The main idea is that cell damage depends on the local DSB density within its nucleus, regardless of the particle type producing it. In this work the input LEM LQ tables for protons, which are needed for the calculation, are obtained from a re-implementation of the LEM by our team [18]. All following references to the ‘LEM’ in this work refer to this re-implementation of the original LEM model. As a benchmark and validation of the capabilities of the LEM re-implementation, comparisons have been made with experimental data for mono-energetic H and He beams for the irradiation of different cell lines [18].

The LQ parameters for protons at different energies are calculated applying the low dose approximation [19], which describes how to link the input LEM-calculated intrinsic proton microscopic parameters, *α*_*z*_ and *β*_*z*_, to the macroscopic dose ones, *α* and *β*. The parameter *α*_*z*_for proton beams at different energies is calculated applying the HIT LEM re-implementation while *β*_*z*_ is calculated as in [20]:

12 $$ {\beta}_z=\frac{S_{\max }-{\alpha}_z}{2{D}_t}. $$

*D*_*t*_ represents the transition dose at which the survival curve for photon irradiation is assumed to have an exponential shape with the maximum slope *S*_max_ = *α*_*x*_ + 2*β*_*x*_*D*_*t*_. Two different values of *D*_*t*_ have been chosen to assess its influence (see Table 1). The *D*_*t*_ values used to test the re-implementation in [18] were low values (≤10.5 Gy); however we have decided to use a large value of *D*_*t*_, 40 Gy, for generating upper limit predictions.

**Table 1:** Photon parameters used for the two representative tissues for low and high (*α/β*)_*x*_

*α*_*x*_ [Gy^-1^]	*β*_*x*_ [Gy^-2^]	(*α*/*β*)_*x*_ [Gy]	*D*_*t*_ [Gy]
0.123	0.0616	2	10; 40
0.616	0.0616	10	10; 40

The other LEM input parameters (cell nucleus area and volume, domain size, etc.) for generating *α* and *β* tables are the same ones as reported in [18]. The *α* and *β* macroscopic parameters of proton beams are used in the Monte Carlo (MC) code for performing LEM-based biological calculations, as described in the next section.

### Monte Carlo implementation

The MC calculations presented in this work have been performed using a FLUKA [21, 22]-based MC framework capable to perform calculations on computed tomography (CT) data with a detailed model of ion irradiation as developed at the Heidelberg Ion Beam Therapy Center (HIT) [23]. The framework includes the modeling of the beam line elements and the patient CT in the FLUKA geometry and a tool for importing the fluences of pencil-like ion beams of different energy and position, as specified in the treatment plan, and for simulating their scanned beam delivery [23]. The MC computational framework has been thoroughly validated and fine-tuned to reproduce dosimetric measurements for HIT treatment conditions [23]. Realistic treatment plans used in this work were prepared at the HIT facility (Syngo-PT Siemens) and then recalculated with the FLUKA version 2011.2b.3. LET_*D*_, absorbed dose and RBE/RBE-weighted dose (*D*_RBE_) calculations based on LEM were performed during runtime following the approach described in [23]. For the biological calculations applying the WED and the CAR model we have converted MC-generated LET_*D*_ maps in RBE/ *D*_RBE_ distributions using the expressions previously introduced, implemented in a post-processing script merging the several outputs of the parallel MC simulations.

Two brain tumor cases and a SOBP in water have been simulated. For the SOBP, a single-field irradiation plan optimized to achieve a homogeneous three-dimensional dose distribution of 2 Gy (RBE) (with a constant RBE of 1.1) in the target region, simulating a 150 mm × 90 mm × 40 mm tumor centered at 76 mm depth, has been calculated with the MC. The FLUKA scoring was performed on 1 × 1 × 1 mm^3^ voxels. In the first patient case (patient 1) a single field enters the skull through the parietal bone and the planning target volume (PTV) is located in the parietal lobe. The second patient (patient 2) is treated by two fields entering the temporal bones in opposite directions. The target volume is located at the base of the skull and several organs at risk are considered by the dose optimization, which was performed using the intensity modulation technique. These cases have been chosen for studying two different scenarios: single field in a homogenous region (patient 1) and two fields irradiation of a heterogeneous region with many critical OARs surrounding the tumor (patient 2). Both plans include 27 identical dose fractions, each delivering 2 Gy (RBE) of proton dose to the PTV assuming a RBE of 1.1. The CT pixel size was 0.65 × 0.65 mm^2^ with a fixed slice thickness of 3 mm. MC distributions were calculated by FLUKA on the CT grid.

### Evaluation

The predictions of the three models have been studied by comparing the *α* and *β* terms of the LQ model as a function of LET, and the RBE values as a function of LET and dose for two tissue types irradiated with proton beams. Parameters characterizing the hypothetical tissues considered for our studies are reported in Table 1. An additional choice of two *D*_*t*_ dose threshold values of 10 and 40 Gy is used. We chose these values for *D*_*t*_ in order to understand the impact of different *D*_*t*_ on the LEM-based biological calculations. However, one could also apply the empirical relationship between (*α*/*β*)_*x*_ and *D*_*t*_ found by Friedrich et al. [24], as for example performed in [14].

Two patient plans have been recalculated analyzing absorbed dose, RBE and *D*_RBE_ distributions for the three models and for the two representative tissues for low and high (*α*/*β*)_*x*_. Dose-volume histograms (DVH), RBE-weighted dose-volume histograms (D_RBE_VH) and dose averaged LET-volume histograms (LET_*D*_VH) have been studied. The effective range variation due to a variable RBE scheme has been assessed by looking at the depth (*R*^RBE^_*x*_) at which the total *D*_RBE_ has decreased to a certain percentage *x* for all depth profiles sampled in beam-eye-view along (each) incidence direction of the single (double) treatment field(s). To account for the fact that several *x* values are typically considered by different facilities, the biological range shift is here calculated as the difference between distal fall-off positions of the physical dose profile multiplied with 1.1 (*R*^RBE = 1.1^) and the *D*_RBE_ profile (*R*^varRBE^) in the beam direction for three percentage *x* values: 90, 80, and 50 % of the prescribed dose (D):

13 $$ {R}_x^{\mathrm{shift}}={R}_x^{\mathrm{varRBE}}-{R}_x^{\mathrm{RBE}=1.1}. $$

This approach basically analyzes the location of the corresponding isodose edge in beam-eye-view, thus quantifying the critical extension of the high dose region to the healthy tissue distal to the tumor along each treatment field incidence direction. Resulting two-dimensional maps of the biological range shift values in beam-eye-view and histograms on the frequency of a certain range shift value among all examined beam directions within the treatment field are evaluated. Profiles that do not reach the dose levels under study are excluded from the analysis. In order to rule out a faulty analysis sensitive to MC statistical fluctuations, only profiles with a steep distal fall-off with a gradient ≤ 30 mm/Gy are considered.

## Results

### Main model dependencies

**Fig. 1:**
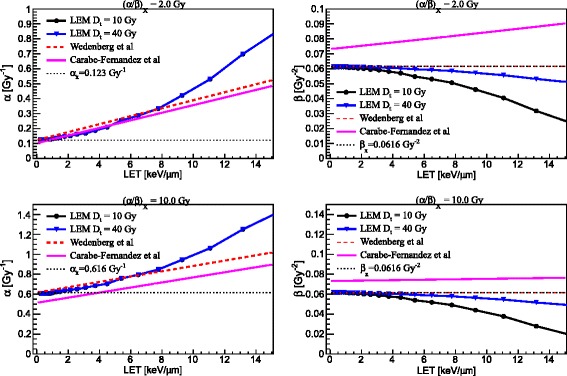
Comparison between three model predictions, as reported in the legends, for *α* (*left column*) and *β* (*right column*) as a function of LET for (*α*/*β*)_*x*_ of photons of 2 Gy (upper panels) and 10 Gy (lower panels). For the LEM, predictions for two values of *D*_*t*_ are reported

**Fig. 2:**
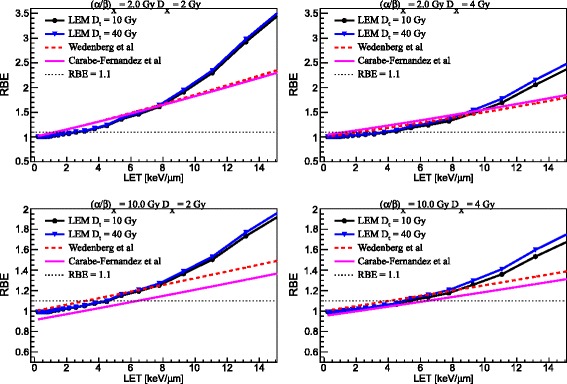
Comparison between three model predictions, as reported in the legends, for RBE as a function of LET for (*α*/*β*)_*x*_ of photons of 2 Gy (*upper panels*) and 10 Gy (*lower panels*) at 2(4) Gy reference photon dose as reported in the left (right) column, respectively. For the LEM, predictions for two values of *D*_*t*_ are reported

**Fig. 3:**
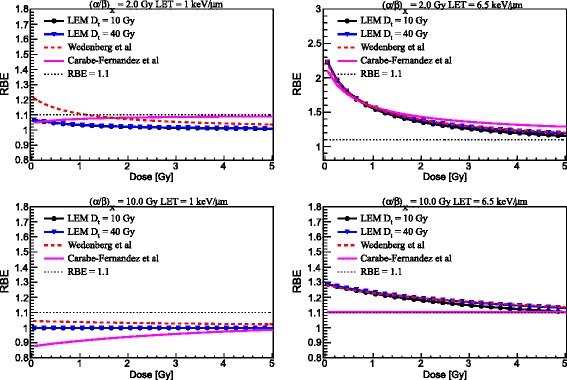
Comparison between three model predictions, as reported in the legends, for RBE as a function of proton dose for (*α*/*β*)_*x*_ of photons of 2 Gy (*upper panels*) and 10 Gy (*lower panels*) at 1(6.5) keV/μm as reported in the left (right) column, respectively. For the LEM, predictions for two values of *D*_*t*_ are reported

Figure 1 shows the comparison between the three models for the prediction of *α* (left panels) and *β* (right panels) for mono-energetic proton beams as a function of proton beam LET for two tissues, as reported in Table 1, with (*α*/*β*)_*x*_ of 2 Gy (upper panels) and 10 Gy (lower panels). RBE results as a function of proton beam LET for the two tissues at two photon dose levels of 2 Gy (left column) and of 4 Gy (right column) are depicted in Fig. 2. Moreover, RBE results at low LET (1.0 keV/μm) and higher LET (6.5 keV/μm) are presented as a function of proton dose for the two tissues in Fig. 3.

### SOBP calculations

**Fig. 4:**
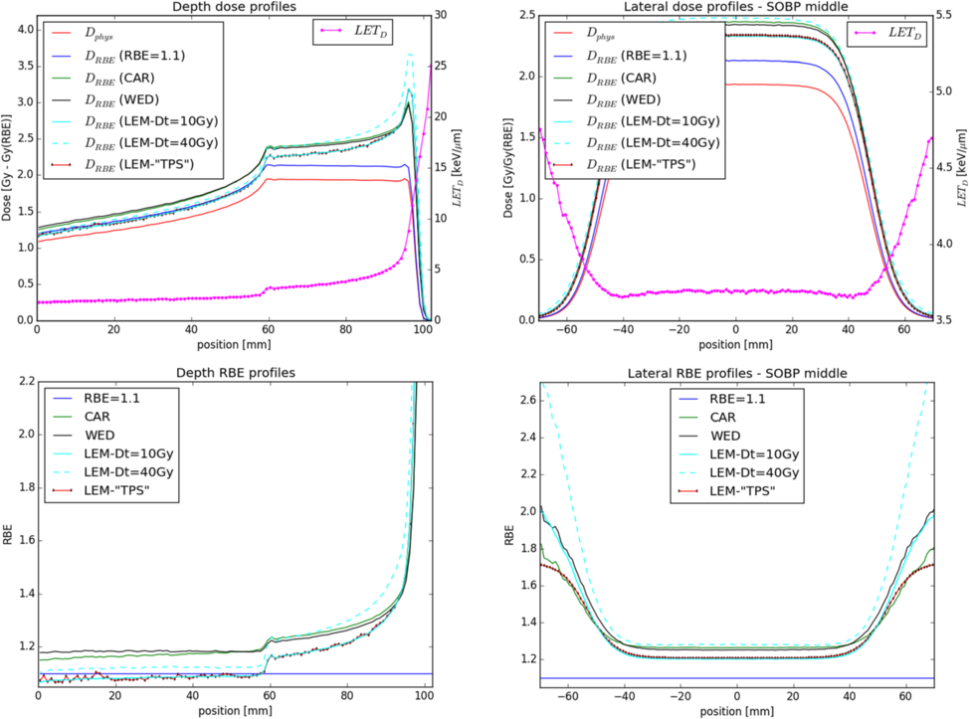
*Left: D*_RBE_ and RBE profiles as function of depth calculated with the three biological models for the (*α*/*β*)_*x*_ = 2 Gy tissue and assuming an RBE of 1.1 are depicted. Dose and LET_D_ values are also shown in the upper panel. *Right:* lateral D_RBE_ and RBE profiles in the middle of the SOBP for the three biological models for the (*α*/*β*)_*x*_ = 2 Gy tissue are shown together with RBE = 1.1 assumption. LET_D_ values are also shown in the upper panel

For comparison of the models in an idealized condition of a SOBP in water, we have calculated depth- and lateral-dose, *D*_RBE_ and RBE profiles applying a tissue with (*α*/*β*)_*x*_ = 2 Gy for a SOBP in water. As an example in the left panels of Fig. 4 dose, LET_*D*_, *D*_RBE,_ and RBE-depth profiles are shown. In order to assess the variation of physical and biological quantities as a function of the lateral distance from the main central axis, D_RBE_, RBE and LET_*D*_ lateral profiles at the middle of the SOBP are depicted in the right panels of Fig. 4. Calculations performed approximating the mixed radiation field composition as being constant regardless of the lateral distance from the central beam axis at each given depth, as applied in our TPS for carbon ion irradiation, is labeled as LEM-“TPS”.

### Patient cases calculations

**Fig. 5:**
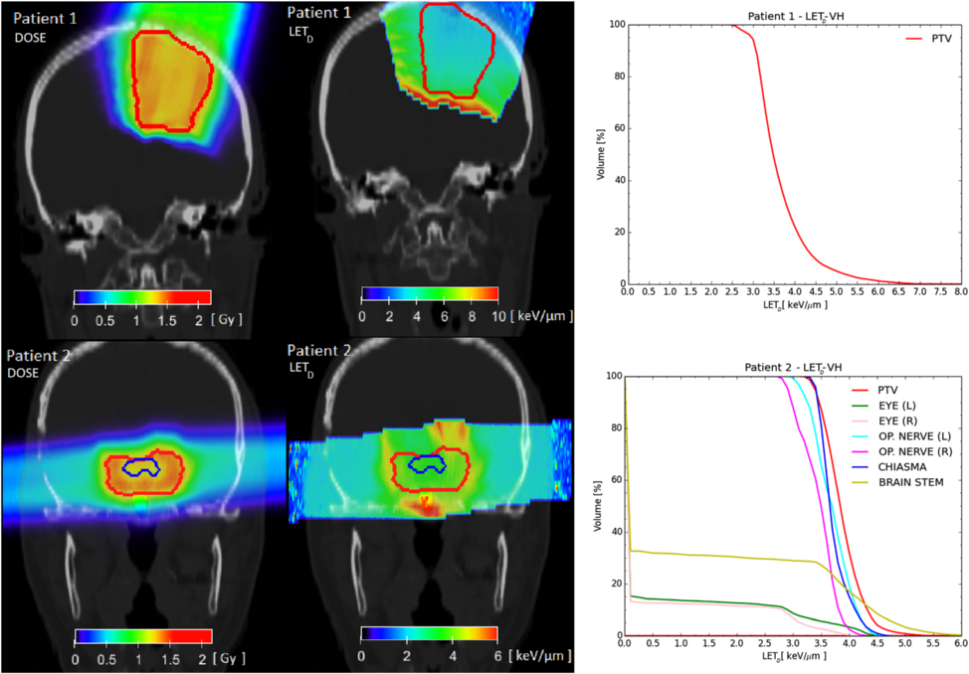
Absorbed dose to water (*left*) and LET_*D*_ (*middle*) distributions for the two patient cases (*top: patient* 1, *bottom: patient* 2) are depicted. PTV and OAR contours are also outlined. LET_*D*_VH are shown in the right panels

**Fig. 6:**
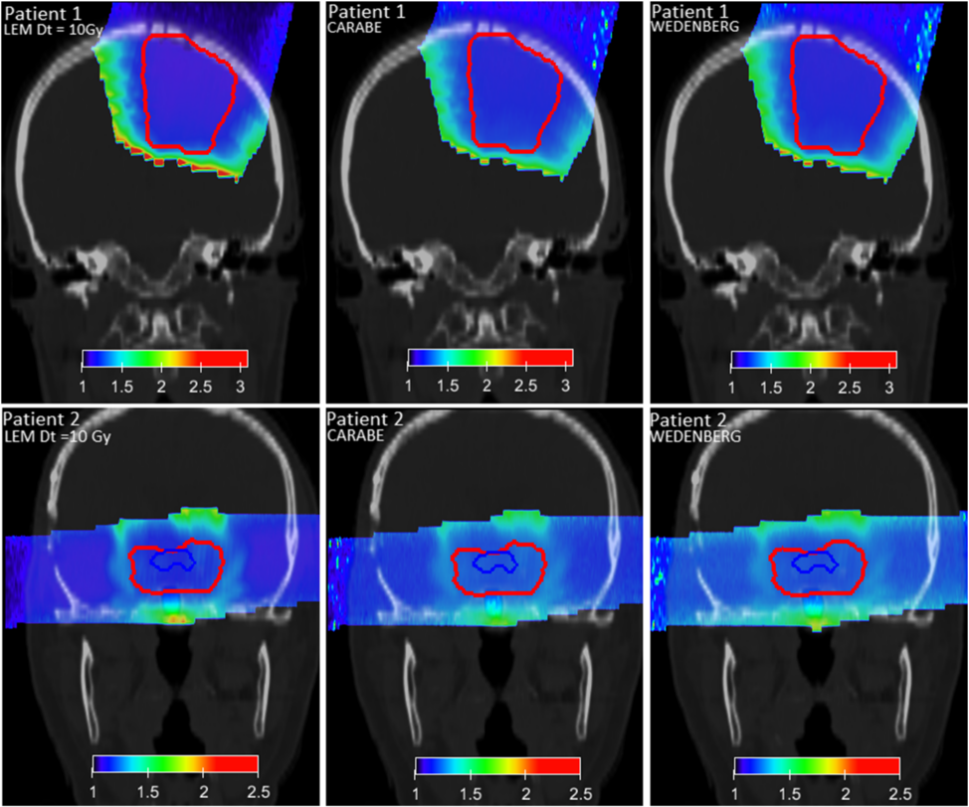
RBE distributions for patient 1 (*upper panels*) and patient 2 (*lower panels*) are shown for (*α*/*β*)_*x*_ = 2 Gy and variable RBE applying the three biological models (left: LEM, middle: Carabe, right: Wedenberg). PTV and OAR contours are depicted with lines

**Fig. 7:**
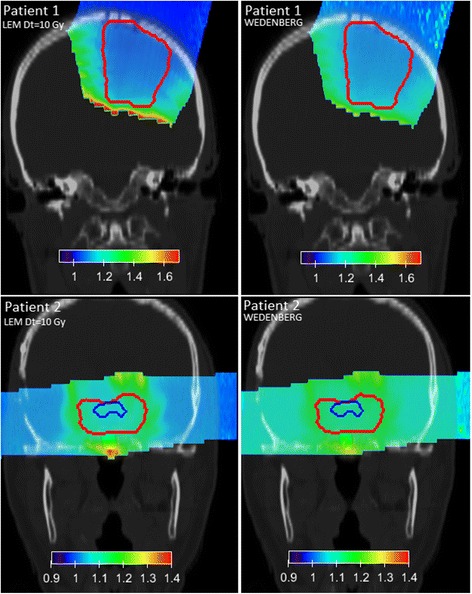
RBE distributions for patient 1 (*upper panels*) and patient 2 (*lower panels*) are shown for (*α*/*β*)_*x*_ = 10 Gy and variable RBE applying the two biological models (*left:* LEM*, right:* Wedenberg). PTV and OAR contours are depicted with lines. The Carabe model is not considered as being outside its range of reliability (cf. Eqs. 14 and 16)

**Fig. 8:**
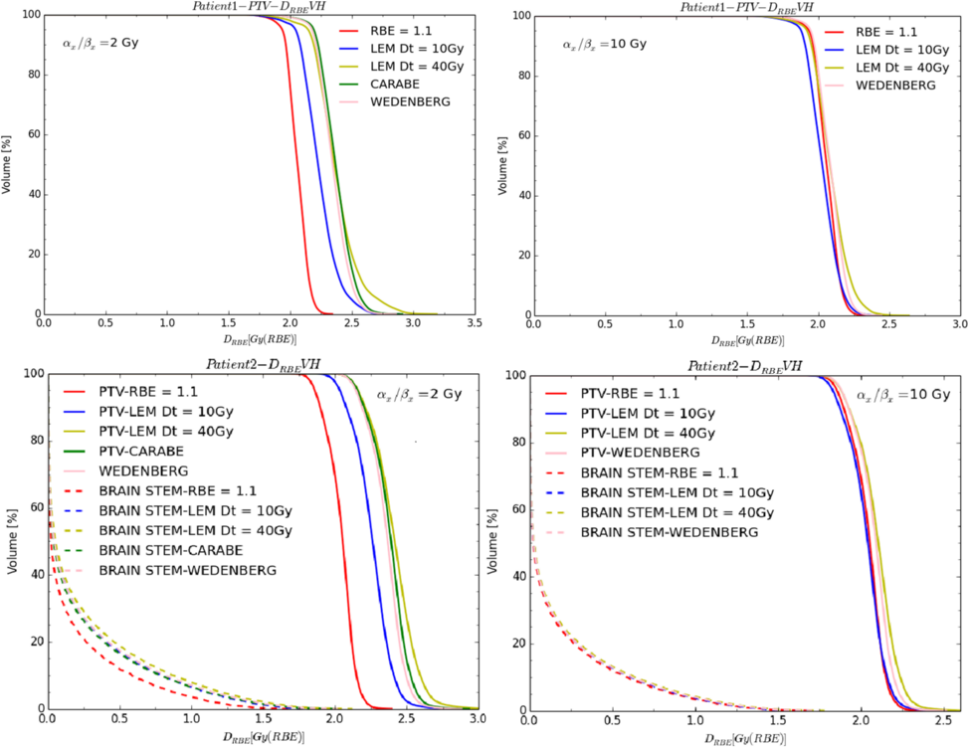
D_RBE_VHs for PTV of patient 1 for (*α*/*β*)_*x*_ = 2(10) Gy are depicted in left (*right*) upper panels for a fixed RBE of 1.1 and variable RBE applying the three (two) biological models. D_RBE_VHs for PTV and brain stem of patient 2 for (*α*/*β*)_*x*_ = 2(10) Gy are depicted in left (*right*) bottom panels for a fixed RBE of 1.1 and variable RBE applying the three (two) biological models

Patient-like treatment cases are summarized in the following. For (*α*/*β*)_*x*_ = 10 Gy the calculations with the CAR model are not included as outside its limit of applicability, as explained in the next section. As an example, in the left and middle panels of Fig. 5 the proton absorbed dose (left) and the LET_*D*_ (right) distributions of the two patients are depicted. PTV and OAR contours are also marked by a line. LET_*D*_VH for the two patient cases are shown in the right panels of Fig. 5. RBE distributions assigning a tissue of (*α*/*β*)_*x*_ = 2 Gy / 10 Gy to the two patients are shown in Figs. 6 and 7, respectively. The resulting minimum (minRBE), maximum (maxRBE) and mean (meanRBE) RBE values in the PTV for the two patient cases are reported in Table 2. D_RBE_VH for the PTV of patient 1 and for the PTV and the brain stem of patient 2 are shown in Fig. 8. The resulting *D*_RBE,95%_, *D*_RBE,5%_ and mean (mean*D*_RBE_) *D*_RBE_ values in the PTV for the two patient cases are summarized in Table 3.

**Table 2:** Minimum (minRBE), maximum (maxRBE) and mean (meanRBE) RBE values in the PTV for the two patient cases for (*α/β*)_*x*_ = 2 Gy and 10 Gy

		(*α*/*β*)_*x*_ = 2 Gy			(*α*/*β*)_*x*_ = 10 Gy		
Patient	Model	minRBE	maxRBE	meanRBE	minRBE	maxRBE	meanRBE
1	LEM *D*_*t*_ = 10 Gy	1.10	1.70	1.19	1.05	1.34	1.09
2		1.17	1.41	1.22	1.05	1.19	1.10
1	LEM *D*_*t*_ = 40 Gy	1.15	1.90	1.27	1.05	1.45	1.12
2		1.22	1.57	1.30	1.09	1.26	1.13
1	WED	1.20	1.64	1.24	1.10	1.26	1.11
2		1.22	1.42	1.27	1.10	1.19	1.12
1	CAR	1.20	1.60	1.27			
2		1.23	1.43	1.28

**Table 3:** *D*_RBE,95 %_, *D*_RBE,5 %_ and mean (mean*D*_RBE_) *D*_RBE_ values expressed in Gy (RBE) in the PTV for the two patient cases for (*α/β*)_*x*_ = 2 Gy and 10 Gy

		(*α*/*β*)_*x*_ = 2 Gy			*α*/*β*)_*x*_ = 10 Gy		
Patient	Model	*D*_RBE,95 %_	*D*_RBE,5 %_	mean*D*_RBE_	*D*_RBE,95 %_	*D*_RBE,5 %_	mean*D*_RBE_
1	RBE = 1.1	1.94	2.19	2.07	1.94	2.19	2.07
2		1.86	2.18	2.04	1.86	2.18	2.04
1	LEM *D*_*t*_ = 10 Gy	2.04	2.50	2.23	1.88	2.20	2.03
2		2.03	2.45	2.26	1.83	2.19	2.03
1	LEM *D*_*t*_ = 40 Gy	2.14	2.71	2.38	1.92	2.29	2.09
2		2.16	2.65	2.41	1.89	2.28	2.10
1	WED	2.17	2.54	2.35	1.95	2.23	2.08
2		2.15	2.53	2.36	1.89	2.23	2.08
1	CAR	2.19	2.57	2.37			
2		2.16	2.57	2.38			

**Fig. 9:**
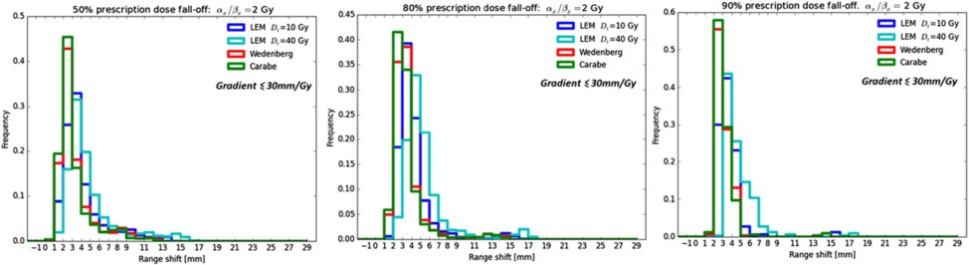
Histograms for biological range shift values for (*α*/*β*)_*x*_ = 2 Gy and patient 1, taking into account profiles exhibiting a gradient ≤ 30 mm/Gy: 90 % *D*_presc_ level in the left panel, 80 % *D*_presc_ level in the middle panel and 50 % *D*_presc_ level in the right panel

As an example of the carried out biological range analysis we report in Fig. 9 the biological range shift values for patient 1 for the (*α*/*β*)_*x*_ = 2 Gy tissue, expressed as frequency histograms. The resulting mean biological range shifts for all analyzed scenarios are reported in Table 4.

**Table 4:** Mean biological range shifts in mm for the two patient cases for (*α/β*)_*x*_ = 2 Gy and 10 Gy as deduced from the beam-eye-view range shift maps

		(*α*/*β*)_*x*_ = 2 Gy			(*α*/*β*)_*x*_ = 10 Gy		
Patient	Model	90 % *D*_presc_	80 % *D*_presc_	50 % *D*_presc_	90 % *D*_presc_	80 % *D*_presc_	50 % *D*_presc_
1	LEM *D*_*t*_ = 10 Gy	3.74	4.33	4.07	1.73	1.81	1.64
2		3.15	2.99	3.55	0.57	0.45	0.66
1	LEM *D*_*t*_ = 40 Gy	4.75	5.42	4.97	2.30	2.44	2.10
2		4.50	4.41	4.84	1.34	1.11	1.22
1	WED	3.16	3.64	3.39	1.11	1.16	1.03
2		3.64	3.60	4.15	0.76	0.64	0.74
1	CAR	3.10	3.51	3.17			
2		3.55	3.37	3.58			

Unreliable shallow profiles (>30 mm /Gy) have been excluded. Three percentage values of the prescription dose have been considered

## Discussion

### Basic features of the models

The variations of *α* and *β* parameters for proton irradiation were shown as a function of LET for the two sets of tissue parameters and for the three models (see Fig. 1). The parameter *α* increases, as expected, with increasing LET in the clinically relevant range for protons, according to all the three models as shown in the left panels of Fig. 1. The LEM and the WED models show *α* to approach *α*_*x*_ at very low LET, whereas the CAR model assumes *α/α*_*x*_ = 0.834 for LET → 0 keV/μm. Nevertheless, for (*α*/*β*)_*x*_ = 2 Gy all models predict quite similar *α* values up to approximately 8 keV/μm. Beyond this value, the LEM exhibits an enhancement in *α*, as described also in [14], that the WED and CAR models do not show. For (*α*/*β*)_*x*_ = 10 Gy *α* increases more slowly but, while the LEM and the WED model show again similar trends, the CAR model predicts a smaller *α*, that is lower than *α*_*x*_ for LET values up to approximately 4 keV/μm. It should be noted that the CAR model has been fitted to experimental data for V79 cells ((*α*/*β*)_*x*_ ≈ 2.7 Gy) and, as a result, it is supposed to be more reliable for a low (*α*/*β*)_*x*_ value than for a high one. No relevant differences for different *D*_*t*_ values are apparent up to 15 keV/μm, for both tissues. Moreover, as discussed in [14], the LEM predicts a vanishing slope for the RBE-LET dependence in the limit of LET → 0 keV/μm, as shown in Fig. 2 of this work.

Trends of the *β* parameter are quite different for the three models (see right panels in Fig. 1). The CAR model predicts *β* to slowly increase as the LET increases, with a slope that decreases with increasing (*α*/*β*)_*x*_ ratio. According to the WED model the parameter *β* is constant and equal to *β*_*x*_, whereas in the LEM framework *β* decreases with increasing LET for the applied LEM implementation. An improved description of the *β* term within the LEM framework can be found in [12]. Moreover, while the LEM predicts *β* to approach *β*_*x*_ for very low LET values, the CAR model assumes *β/β*_*x*_ = 1.09 for LET → 0 keV/μm. Understandably, variations of *D*_*t*_ affect much more *β* than *α* and are more noticeable for high LET values, since *D*_*t*_ is expected to become more important with increasing dose.

Figure 2 shows RBE predictions as a function of the LET for the two considered tissues at two different photon dose levels: 2 and 4 Gy. As expected, the RBE increases for increasing LET in the LET range analyzed, decreases for increasing dose, and increases for decreasing (*α*/*β*)_*x*_ of the tissue.

Despite different trends found for *β* in case of (*α*/*β*)_*x*_ = 2 Gy (see upper panels in Fig. 2), the three models predict similar RBE values for LET values up to 8 keV/μm for both dose values (mainly due to compensation effects between *α* and *β*), whereas beyond 8–10 keV/μm the LEM shows a RBE prediction higher than the other models (due to the enhancement of *α*). The RBE is equal to 1.1 approximately at 2.5 keV/μm for the LEM and between 1 and 1.5 keV/μm for the other models at a dose level of 2 Gy. However, at a dose level of 4 Gy, the RBE reaches the value of 1.1 approximately at 4 keV/μm according to the LEM, at 1 keV/μm for the CAR model, and at 2 keV/μm for the WED model. According to the CAR model, at *D*_*x*_ = 4 Gy the RBE does not approach unity for very low LET values due to the parameter *β* being higher than *β*_*x*_ in the low LET region (and becoming more important at higher doses). For (*α*/*β*)_*x*_ = 10 Gy, as shown in the lower panels in Fig. 2, the LEM and the WED model show a similar trend to each other, while the CAR model predicts the RBE to be smaller than one for low LET values. This is a consequence of *α* being lower than *α*_*x*_ in the low LET region. The effect is reduced at *D*_*x*_ = 4 Gy because of the reduced importance of *α*. The RBE is equal to 1.1 approximately at 3 keV/μm for the WED model, at 4.5 keV/μm for the LEM, and at 6 keV/μm for the CAR model at a dose level of 2 Gy. Conversely, at a dose level of 4 Gy, the RBE achieves the value of 1.1 approximately at 4 keV/μm according to the WED model, at 5 keV/μm for the LEM, and at 6 keV/μm for the CAR model. The RBE predictions by the LEM model when varying *D*_*t*_ differ beyond approximately 5 keV/μm. The difference increases as LET and dose increase.

Figure 3 shows predictions of RBE as a function of the proton dose by the three models for low LET (1 keV/μm) and high LET (6.5 keV/μm). The LEM and the WED model exhibit similar trends. The RBE increases with decreasing dose with only an exception at low LET and high (*α*/*β*)_*x*_ ratio. For (*α*/*β*)_*x*_ = 2 Gy (upper panels in Fig. 3), and low LET, according to the LEM, the RBE is between 1 and 1.1 at any dose level, whereas it is higher than 1.1 for dose values smaller than ≈ 1.2 Gy according to the WED model. At high LET, the RBE is higher than 1.1 in the studied dose range for all the three models, and reaches values between 2 and 2.3 at very low dose values. For (*α*/*β*)_*x*_ = 10 Gy (bottom panels in Fig. 3), at low LET, the RBE increases slightly with decreasing dose according to the WED model, whereas it is almost constant in the studied dose range according to the LEM. At high LET, the RBE is higher than 1.1 in the whole dose range and reaches values between 1.2 and 1.3 at very low doses. Variations in the RBE predictions by the LEM when changing *D*_*t*_ are apparent only for high LET and at high dose. The CAR model predicts that for high LET the RBE decreases as the dose increases for (α/β)x = 2 Gy while for (α/β)x = 10 Gy it remains nearly constant. Moreover, the CAR model predicts the RBE to increase with increasing dose, at least for low LET and in particular for (*α*/*β*)_*x*_ = 10 Gy. This is due to the fact that RBE_max_ can be lower than RBE_min_ under certain conditions. In fact, expressing Eq. (4) as a function of the proton dose instead of the photon dose (see for example [9]):

$$ \mathrm{R}\mathrm{B}\mathrm{E}\left[{\left(\alpha /\beta \right)}_x,D,{\mathrm{RBE}}_{\max },{\mathrm{RBE}}_{\min}\right]=-\frac{1}{2D}{\left(\frac{\alpha }{\beta}\right)}_x+\frac{1}{D}\sqrt{\frac{1}{4}{\left(\frac{\alpha }{\beta}\right)}_x^2+RB{E}_{\max }{\left(\frac{\alpha }{\beta}\right)}_xD+RB{E}_{\min}^2{D}^2}, $$

its derivative^1^ with respect to the proton dose results to be negative only if RBE_max_ > RBE_min_:

$$ \frac{\mathrm{d}}{\mathrm{d}\kern0.1em D}\mathrm{R}\mathrm{B}\mathrm{E}\left[{\left(\alpha /\beta \right)}_x,D,{\mathrm{RBE}}_{\max },{\mathrm{RBE}}_{\min}\right]<0\kern1em \Rightarrow \kern1em {\mathrm{RBE}}_{\max }>{\mathrm{RBE}}_{\min }, $$

namely:

14 $$ \Rightarrow \kern1em \frac{{\mathrm{LET}}_D}{{\left(\alpha /\beta \right)}_x}>0.62\;\left[\frac{\mathrm{keV}/\upmu \mathrm{m}}{\mathrm{Gy}}\right]\;. $$

If Eq. (14) is not fulfilled the CAR model should be considered inapplicable. As a consequence, for the tissues studied in this work, the CAR model can only be considered applicable if:

15 $$ \mathrm{LET}>1.24\;\left[\mathrm{keV}/\upmu \mathrm{m}\right]\kern1em \mathrm{f}\mathrm{o}\mathrm{r}\kern0.5em {\left(\alpha /\beta \right)}_x=2\;\mathrm{Gy}, $$

16 $$ \mathrm{LET}>6.20\;\left[\mathrm{keV}/\upmu \mathrm{m}\right]\kern1em \mathrm{f}\mathrm{o}\mathrm{r}\kern0.5em {\left(\alpha /\beta \right)}_x=10\;\mathrm{Gy}\;. $$

For this reason, the CAR model has not been taken into account for the considered clinical investigations with (*α*/*β*)_*x*_ = 10 Gy. However, the results presented in [25] suggest that the condition RBE_max_ > RBE_min_ could, in certain cases, not be fulfilled, i.e., RBE at low doses of low LET particles (e.g., low LET carbon ions in the Karger and collaborators’ paper) is lower than the RBE for high doses of the same particles with the same LET. Additional experimental investigations are needed for further understanding this scenario and in general the capabilities and the limits of the models analyzed.

### Model dependencies in a SOBP

*D*_RBE_ and RBE profiles as function of depth calculated with the three biological models for the (*α*/*β*)_*x*_ = 2 Gy tissue and assuming an RBE of 1.1 are depicted in Fig. 4 (left panels) together with the dose and LET_*D*_ values (upper-left panel).

The CAR and WED models produce similar *D*_RBE_ values always higher than the clinically assumed one (with a RBE of 1.1). The CAR/WED RBE values in the entrance and in the middle of the SOBP are, respectively, 1.15/1.18 and 1.27/1.26. The LEM *D*_RBE_ prediction assuming *D*_*t*_ = 10 Gy is close to the *D*_RBE_ values with RBE = 1.1 in the entrance channel, while it increases as function of depth in the high dose region of the SOBP, eventually exceeding the CAR and the WED predictions in the last millimeter of the SOBP. Applying higher *D*_*t*_ values produces an enhancement of the *D*_RBE_ at the depths with higher LET_*D*_ values. LEM RBE values in the entrance and in the middle of the SOBP are respectively, 1.07/1.1 and 1.21/1.29 for *D*_*t*_ = 10 Gy / 40 Gy.

Lateral *D*_RBE_ and RBE profiles in the middle of the SOBP for the three biological models for the (*α*/*β*)_*x*_ = 2 Gy tissue are depicted in the right panels of Fig. 4, together with the LET_*D*_ values.

Analyzing the lateral *D*_RBE_ profiles in terms of 80 – 20 % fall-off we have found the following values: 13.3 mm with RBE 1.1, 13.9 mm for LEM-“TPS” approximation, 14.0 mm for the WED model, 13.9 mm for the CAR model and 14.1/14.7 for the LEM with *D*_*t*_ = 10 Gy / 40 Gy. Hence, a widening of the field in terms of D_RBE_ of roughly 1.0 mm in comparison to assuming a constant RBE of 1.1 has been found independent of the model used (CAR/WED/LEM). Properly taking into account the variation of the mixed radiation field (secondary charged particles produced in nuclear reactions) not only as a function of depth, but also laterally, results in an increase of the RBE in the low dose region (comparing LEM and LEM-“TPS” like predictions) and an increase in the lateral fall-off of about 0.2 mm. This region corresponds to higher LET_*D*_ values compared to the central part of the field, due to the primary protons and the secondary higher LET particles stopping.

### Model dependencies in patient cases

The aim of proton treatment planning is to deliver a dose as uniform as possible to the target, sparing healthy tissues (see Fig. 5). Uniform dose distributions do not ensure a homogeneous LET_*D*_ distribution. Moreover, equivalent dose distributions do not necessarily correspond to equivalent LET_*D*_ distributions. High LET_*D*_ regions can be distributed in a complex way in a patient geometry, especially if more than one field is applied. Representative slices of LET_*D*_ distributions and the LET_*D*_VH of the two patients are shown in Fig. 5.

For patient 1, there is a region of intermediate LET_*D*_ values (2.5–5 keV/μm), covering almost the whole PTV (95 %) and in the remaining volume values up to 8.1 keV/μm are observed. In the region posterior to the target (with respect to the beam direction) the LET_*D*_ is between 8 and 12 keV/μm.

For patient 2, LET_*D*_ is between 3 and 4.5 keV/μm in almost the entire PTV (95 %), and it is up to 6.5 keV/μm in the remaining volume. For optic chiasma and nerves, which are partially included in the PTV, LET_*D*_ values do not exceed 5 keV/μm. Higher LET_*D*_ spots are located in tissues surrounding the PTV, e.g., 5 % of the brain stem volume exhibits LET_*D*_ values beyond 5 keV/μm and a maximum LET_*D*_ of 7.4 keV/μm. LET_*D*_ values found in this study are consistent with the values found in [26].

Taking into account LET_*D*_ conditions on the LET values for the CAR model reported above, one can conclude that the CAR approach can be applied for RBE/*D*_RBE_ calculations only for (*α*/*β*)_*x*_ = 2 Gy in our case.

In Figs. 6 and 7, RBE distributions obtained from the three (two) models for (*α*/*β*)_*x*_ = 2(10) Gy are shown, respectively, for the two patients.

For patient 1, the three models predict the RBE to be between 1.1(1.05) and 1.9(1.5) in the PTV for (*α*/*β*)_*x*_ = 2(10) Gy, respectively (see Table 2).

For patient 2, the three models predict the RBE to be between 1.2 and 1.6 in the PTV (see Table 2). High RBE spots correspond, as expected, to high LET_*D*_ values. For (*α*/*β*)_*x*_ = 10 Gy, the RBE varies more slowly with increasing LET. These results are in line with the values found in a previous publication [7]. There, the authors found RBE values between 1.1 and 1.2 within the clinical target volume in a multiple field hypopharinx case ((*α*/*β*)_*x*_ ≈ 10 Gy) and higher values in the spinal cord ((*α*/*β*)_*x*_ ≈ 2 Gy). Moreover, our results are in agreement with values found by Gerweck and Kozin in [27] for (*α*/*β*)_*x*_ ≈ 7–13 Gy in cell survival experiments.

Representative D_RBE_VHs are shown in Fig. 8 for the two patient cases. For patient 1 in case of (α/β)_x_ = 2 Gy, when applying RBE = 1.1, a *D*_RBE_ between 1.9 and 2.2 Gy (RBE) is obtained in the PTV. Conversely, applying the three models yields *D*_RBE_ values between 2.0 and 2.7 Gy (RBE) (see Table 3). CAR, WED and LEM with *D*_*t*_ = 40 Gy give similar mean*D*_RBE_ values, while LEM with *D*_*t*_ = 10 Gy estimates an about 6 % lower mean*D*_RBE_ value inside the PTV.

*D*_RBE_ predictions for patient 1 for (*α*/*β*)_*x*_ = 10 Gy by all the models are consistent (within about 3 % looking at mean*D*_RBE_) and similar to the *D*_RBE_ obtained with a fixed RBE of 1.1. Applying the LEM with *D*_*t*_ = 10 Gy or 40 Gy seems to have a low impact on D_RBE_VH PTV for (*α*/*β*)_*x*_ = 10 Gy (see Table 3).

For patient 2, larger variations between fixed and variable *D*_RBE_ have been found for (*α*/*β*)_*x*_ = 2 Gy with respect to (*α*/*β*)_*x*_ = 10 Gy, especially within the PTV. The PTV exhibits *D*_RBE_ values between 1.9 and 2.2 Gy (RBE) for RBE = 1.1 and between 2.0 and 2.7 Gy (RBE) for variable RBE, in the case of (*α*/*β*)_*x*_ = 2 Gy (see Table 3). For the brain stem, with the (*α*/*β*)_*x*_ = 2 Gy tissue, the *D*_RBE,5%_ values range from 0.9 Gy (RBE) applying RBE = 1.1 up to 1.2 Gy (RBE) for variable RBE.

In summary, *D*_RBE_ predictions by the three considered models are often higher than the values used in clinics with a fixed RBE of 1.1, especially for high LET_*D*_ areas and low (*α*/*β*)_*x*_ ratios. Variations exceeding 10 % of the prescription dose were found within the PTV. Since 5 % dose variations can produce 10–20 % variations in tumor control probability and 20–30 % variations in normal tissues complication probability [28], differences found in this study can be clinically significant. However, it should be kept in mind that lower RBE values are typically found in vivo compared to the in vitro ones, which are inherently affecting our model calculations.

It is interesting to notice that similar variations were also observed when using the more recent model of McNamara et al. [29], which draws on very similar assumptions as the WED model for the α term, but on a more recent re-evaluation of in-vitro proton experiments reported in [3]. In this case, agreement within about 6 % in terms of mean D_RBE_ in the PTV was observed with the other LET_D_-based models.

In terms of range variations, Fig. 9 shows, as an example, biological range shift values reported as histograms for patient 1, when using the (*α*/*β*)_*x*_ = 2 Gy tissue. Mean biological range shifts for the two patients and the two tissues are reported in Table 4. Carabe and collaborators found similar range shift values (2–3 mm) for a (*α*/*β*)_*x*_ approximately equal to 2 Gy, applying the CAR model to a patient case [4]. Moreover, increasing the *D*_*t*_ value for LEM produces on average larger biological range shifts. All presented results suggest caution in proton therapy treatment planning, especially if OARs are close to the target. Usually, safety margins are applied to take into account range uncertainties and to ensure the target dose coverage.

The observed findings suggest that, regardless of the used biological model, a biological range shift margin of few millimeters distal to the target volume (with respect to the chosen beam direction) should be taken into account when designing the treatment [30]. Since safety margins are calculated with different procedures at each facility [31], findings comparing the outcome of different models, as performed in this study, may help developing some general recommendations for medical physicists during treatment planning. For example, opposite beams arrangements, when clinically available, should be preferred to single field/orthogonal ones to reduce the resulting LET_*D*_ and RBE values, as shown comparing the patient cases in this work. Moreover, active beam scanning techniques should be further exploited to push LET_*D*_ areas away from OARs, while preserving dose coverage in the PTV. The PTV definition should take into account biological range uncertainties. In addition to obvious depth dependences of LET variations, also lateral variations due to scattering effects should not be neglected and could become important at the tumor edges, as more clearly shown by the SOBP study in water (Fig. 4 - right panel).

## Conclusions

Despite using models with quite different assumptions, the observed results show largely consistent deviations to the current practice of proton therapy biological planning with a constant RBE of 1.1. These findings suggest that it is worth considering at least main RBE dependencies (dose, LET and (*α*/*β*)_*x*_) in treatment planning, especially if beams point toward or pass laterally adjacent to OARs, and being particularly cautious for tissues with a low (*α*/*β*)_*x*_ ratio. Hence, it is strongly advisable to have computational tools which can model such variable RBE (maybe even using simpler phenomenological models such as the WED model or the more recent McNamara et al. model) and use those variations as general guidance in taking biological uncertainties into account. Future work should indeed provide better experimental insights to enable identification of the model of choice, including a comparison to more recent and not yet thoroughly examined models such as the McNamara et al., the RMF and the MKM models, and the underlying optimal parameters for eventual clinical deployment towards direct planning, besides the suggested usage for robustness assessment.

## Abbreviations

CAR model: Carabe-Fernandez model
CT: computed tomography
D_RBE_: RBE-weighted dose
D_RBE_VH: RBE-weighted dose-volume histogram
DSB: double strand breaks
DVH: dose-volume histogram
HIT: Heidelberg ion beam therapy center
LEM: local effect model
LET: linear energy transfer
LET_D_: dose-averaged linear energy transfer
LET_D_VH: dose-averaged LET-volume histogram
LQ model: linear quadratic model
MC: Monte Carlo
MKM: microdosimetric kinetic model
OAR: organ at risk
PTV: planning target volume
RBE: relative biological effectiveness
RMF: repair misrepair fixation
SOBP: spread out Bragg peak
TPS: treatment planning system
WED model: Wedenberg model

## Footnotes

1 The derivative can be performed with the tools available on-line such as *Wolfram|Alpha.*
